# Interfacing DNA nanotechnology and biomimetic photonic complexes: advances and prospects in energy and biomedicine

**DOI:** 10.1186/s12951-022-01449-y

**Published:** 2022-06-03

**Authors:** Xu Zhou, Su Lin, Hao Yan

**Affiliations:** 1 Center for Molecular Design and Biomimetics at the Biodesign Institute, Arizona State University, Tempe, AZ 85287 USA; 2 School of Molecular Sciences, Arizona State University, Tempe, AZ 85287 USA

**Keywords:** DNA nanotechnology, Dye aggregates, Energy transfer, Biosensing, Bioimaging, Cancer therapy

## Abstract

Self-assembled photonic systems with well-organized spatial arrangement and engineered optical properties can be used as efficient energy materials and as effective biomedical agents. The lessons learned from natural light-harvesting antennas have inspired the design and synthesis of a series of biomimetic photonic complexes, including those containing strongly coupled dye aggregates with dense molecular packing and unique spectroscopic features. These photoactive components provide excellent features that could be coupled to multiple applications including light-harvesting, energy transfer, biosensing, bioimaging, and cancer therapy. Meanwhile, nanoscale DNA assemblies have been employed as programmable and addressable templates to guide the formation of DNA-directed multi-pigment complexes, which can be used to enhance the complexity and precision of artificial photonic systems and show the potential for energy and biomedical applications. This review focuses on the interface of DNA nanotechnology and biomimetic photonic systems. We summarized the recent progress in the design, synthesis, and applications of bioinspired photonic systems, highlighted the advantages of the utilization of DNA nanostructures, and discussed the challenges and opportunities they provide.

**Graphical Abstract:**

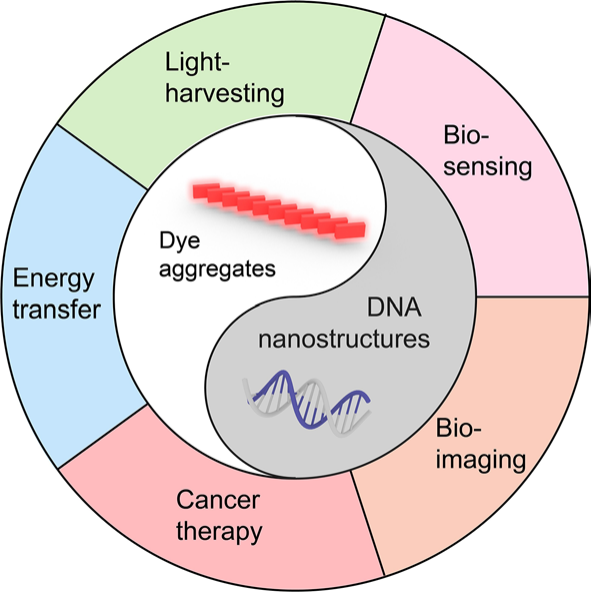

Light is a critical energy source and a powerful tool for the manipulation of matter. The interaction of light and matter can be engineered via tuning the structures and properties of optical materials for desired functions and applications in multiple fields including energy, environment, biomedicine. In this review, we focused on the interface of biomimetic photonic systems, particularly self-assembled multi-chromophore complexes, and structural DNA nanotechnology. We summarized the development of multi-pigment complexes and their applications in energy and biomedicine for light-harvesting, sensing, imaging, and therapy. Approaches for the employment of DNA nanostructures to direct the assembly of photonic systems and their potential as a novel class of energy and biomedical materials are also discussed, along with an emphasis on the challenges and opportunities this class of materials provides.

## Nature-inspired functional multichromophore complex

Rational design and synthesis of photoactive systems that exhibit desired optical properties and robust capabilities for photon capture and energy conversion are in great demand for their potential applications in energy and biomedicine, and nature has provided a blueprint for addressing these challenges. Specifically, photosynthetic organisms use an exquisite molecular arrangement of photosynthetic pigments in light-harvesting antenna systems for photon capture and energy transfer. Studies of the molecular structure and mechanism for these complexes reveal general principles that can be applied to guide the design of biomimetic photonic systems for energy and biomedical applications.

### Natural light-harvesting system

Photosynthesis is one of the most important biological activities on our planet. Photosynthetic species capture photons from sunlight and store the solar energy with chemical potential. The process is initiated by efficient light-harvesting and the absorbed excitation energy is further transferred to the reaction center where the energy of the photon is converted to the chemical energy which catalyzes a series of dark reactions. The photon capture and energy transfer takes place in light-harvesting antenna systems that present a high degree of structural diversity due to the adaptive evolution of photosynthetic organisms in a vast array of diverse environments [1]. Light-harvesting complexes are sophisticated pigment-protein complexes that are organized by protein scaffolds arranged with precise position and orientation. Chromophores can be divided into three groups: chlorophylls, carotenoids, and phycobilins, all of which show specific spectral features and high extinction coefficients due to their different conjugated pi-electronic structures [2]. The protein scaffolds control the spatial arrangement as well as environment and dynamics of various pigments to eliminate the possibility for self-quenching, and create a downhill energy landscape for guiding absorbed energy to the reaction center with high efficiency [3].

**Fig. 1:**
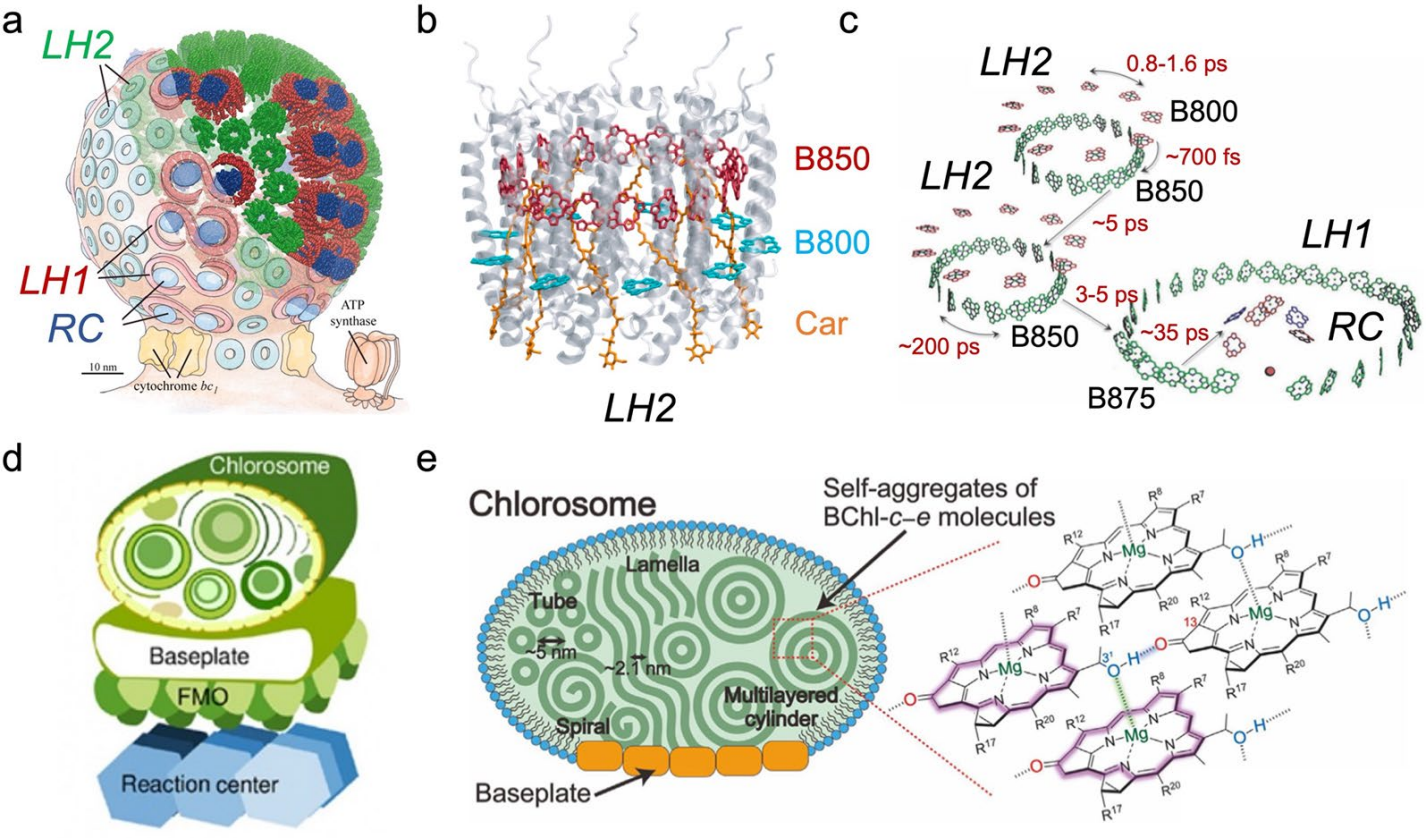
Examples of natural light-harvesting systems. **a** Structure of the photosynthetic unit of purple non-sulfur bacteria. **b** Model of the light-harvesting complex 2 (LH2). **c** Energy transfer in the network formed by the light-harvesting complex1 (LH1) and LH2 structures. **d** Model of the photosynthetic unit of green sulfur bacteria. **e** Model of the chromosome and the molecular arrangement of self-aggregated chromophores. Reprinted with permission from: **a** Ref. [9], copyright (2007) National Academy of Sciences, U.S.A; **b** Ref. [1], copyright (2017) American Chemical Society; **c** Ref. [5], copyright (2012) Royal Society of Chemistry; **d** Ref. [12], copyright(2014) American Chemical Society; **e** Ref. [13], copyright (2014) Elsevier

In certain photosynthetic light-harvesting systems, the delocalized excitation state is observed [1, 4, 5]. This occurs when the chromophores are densely packed causing strong intermolecular electronic interactions that result in a shared excitation state among multiple molecules called molecular excitons. The light-harvesting complex 2 (LH2) from purple non-sulfur bacteria *Rps. acidophila* is one example [6, 7], whereby LH2 exhibits two ring-like structures B800 and B850 assembled by bacteriochlorophyll-a (BChl-a) molecules (Fig. 1a, b). In the B800 ring, BChl-a molecules are aligned with a center-to-center distance of ~ 21 Å, and the weak inter-chromophore coupling results in a localized excitation state. Contrary to its counterpart, the B850 ring is formed by a set of 18 densely spaced BChl-a molecules with a center-to-center distance of ~ 9 Å, causing a strongly coupled intermolecular interaction which induces a delocalized exciton within the ring structure resulting in a shift in absorption from 800 nm for BChl-a molecule to 850 nm. The LH2 and light-harvesting complex 1 (LH1) further assembles into a large-scale interconnected network, in which the captured excitation energy can move freely among the entire network for efficient light collection and transport (Fig. 1c) [8, 9]. Another example is the chlorosome, which is a peripheral light-harvesting antenna complex from green sulfur bacteria which contains ~ 200,000 bacteriochlorophylls (BChl) that are self-aggregated to multiple rod-like structures and enclosed by the lipid monolayer as “sacks” [10–12]. (Fig. 1d). These aggregated BChl complexes are closely packed and exhibit strongly coupled excitonic features [13], (Fig. 1e) and a delocalized excitation state capable of tuning the spectral features to facilitate efficient energy transfer [3, 4].

### Molecular exciton and dye aggregates

**Fig. 2:**
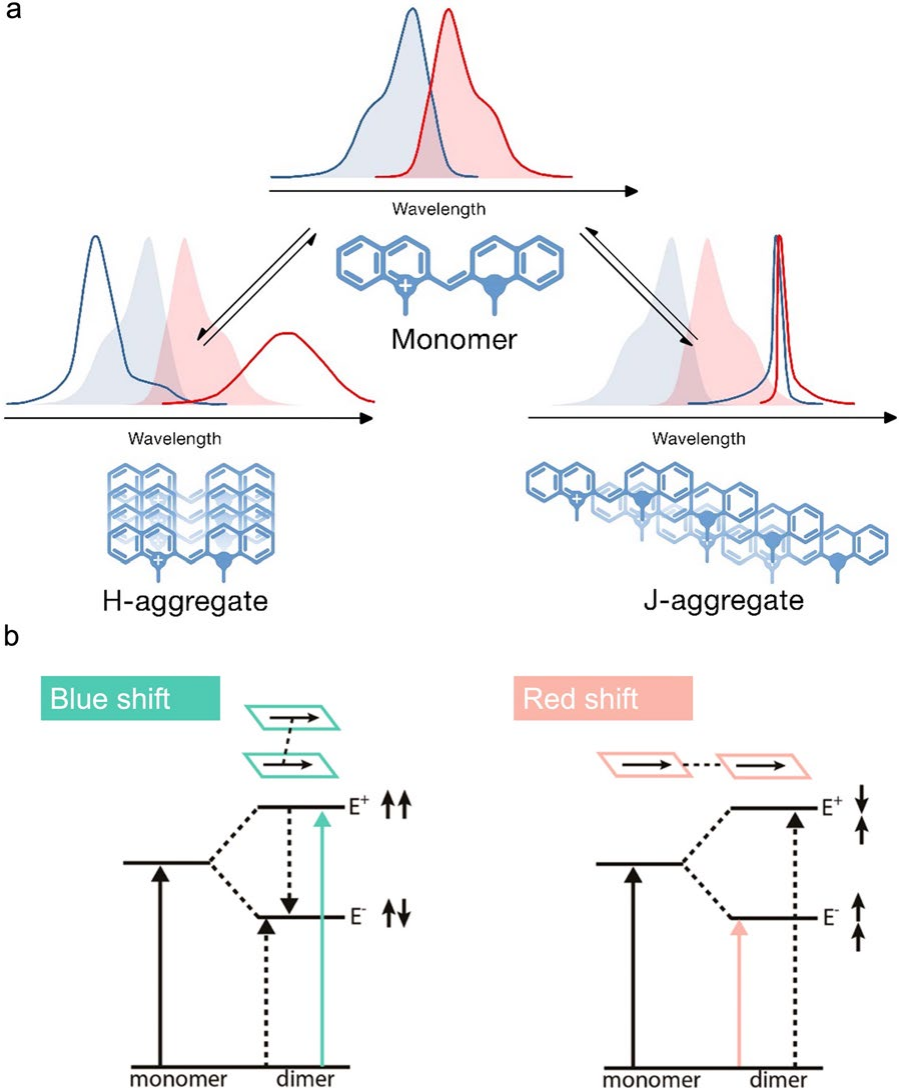
Spectral features and molecular arrangements of J- and H-aggregates. **a** Model of spectra and molecular arrangements for J- and H-aggregates. **b** Excited state energy diagrams of molecular dimers with two different arrangements (left: H-dimer, right: J-dimer). Reprinted with permission from: **a** Ref. [15], copyright (2014) IOP Publishing; **b** Ref. [1], copyright (2017) American Chemical Society

The molecular exciton is characterized by its delocalized excitation state that results from strong interactions of the chromophores. In this case, the excitation state is coherently shared by more than one molecule rather than isolated on individual molecules. In addition to the natural light-harvesting systems, the delocalized exciton is also observed in self-assembled synthetic dye aggregate systems. Dye aggregates with highly ordered and closely packed molecular arrangements can be classified into two forms: J-aggregates (J denotes Jelley, who initially characterized the aggregate) and H-aggregates (H denotes hypsochromic) based upon their unique spectroscopic characteristics [14, 15]. (Fig. 2a) J-aggregates typically include features such as largely shifted absorption and emission spectra to a longer wavelength (red-shifted) compared with the monomeric chromophore, a narrow absorption band (J-band), an enhanced emission band with a small Stokes shift, and a shorter fluorescence lifetime called ‘superradiance’, compared with the monomer. By contrast, H-aggregates exhibit a hypsochromically shifted absorption band (shifted to a shorter wavelength) and a reduced fluorescence emission intensity.

The difference in optical properties of J- and H-aggregates is attributed to their unique molecular arrangements. In J-aggregates, the constituent chromophores are aligned in a ‘head-to-tail’ arrangement, whereas in H-aggregates, the pigment molecules are assembled ‘side-by-side’ with sandwich-like geometry [15]. A molecular exciton model has been applied in both scenarios [16, 17]. A dimeric molecular assembly (AB) is one example, where the delocalized excitation state can be described as a linear combination of the excited A (A*B) and the excited B (AB*) to give two excited energy levels that are separated by the interaction energy between the two component molecules (ΔE) (Fig. 2b). When the two molecules are arranged ‘head-to-tail’, only the transition to the lower energy level is allowed, consistent with the bathochromic spectral features of J-aggregates. Alternatively, the ‘side-by-side’ molecular arrangement results in a favorable transition to the higher excited level, which is consistent with the hypsochromic absorption spectra of H-aggregates.

### Artificial dye materials to harness and collect photons

**Fig. 3:**
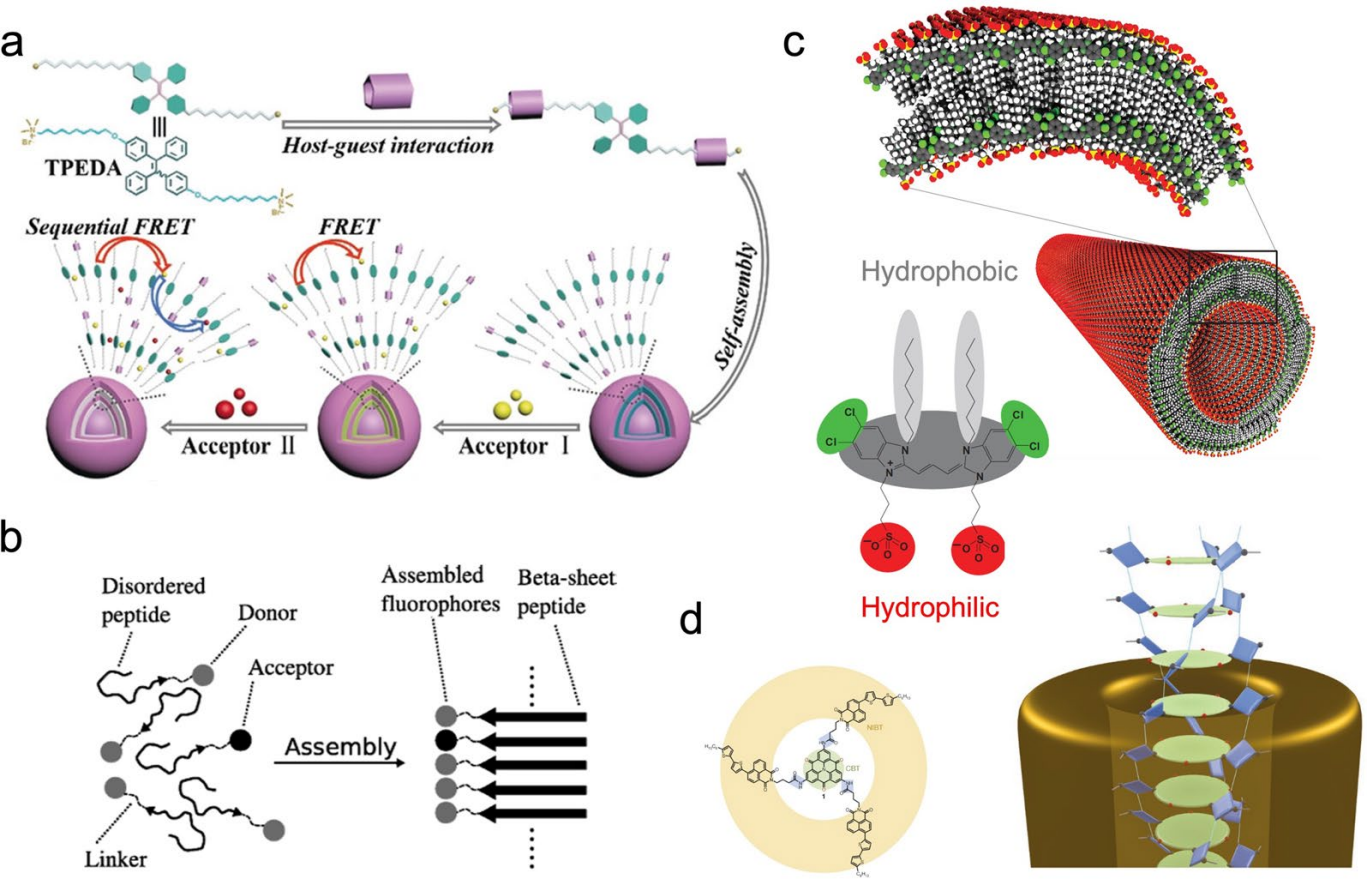
Bio-inspired artificial light-harvesting systems. The artificial light-harvesting systems are either organized by **a** guest–host assemblies [27] and **b** self-assembled peptide structures [23], or formed by **c** self-assembled dye aggregates nanotubes[28] and **d** nanofibers[31]. Reprinted with permission from: **a** Ref. [27], copyright (2020) Wiley; **b** Ref. [23], copyright (2009) American Chemical Society; **c** Ref. [28], copyright (2009) Springer Nature; **d** Ref. [31], copyright (2015) Springer Nature

Natural light-harvesting systems can be used to inspire the rational design of artificial light-harvesting complexes for efficient photon collection and exciton transport because of their ability to organize multi-pigment systems at high density using protein scaffolds to generate a specific energy landscape for efficient excitation energy transport along a defined pathway [1, 2]. Similarly, artificial excitonic architectures can be constructed by employing natural or synthetic scaffolds to organize dye molecules for the formation of multi-chromophore complexes with coupled intermolecular interactions. A variety of scaffolds have been reported including viral capsid proteins [18], covalent dendrimers [19], supramolecular micelles [20] and nanotubes [21], metal–organic frameworks [20–22], self-assembled peptide [23, 24] and protein [25] structures, DNA nanostructures[26], and others. Wang et al. employed a host–guest complex that loaded two fluorescent dyes, Eosin Y and Nile Red, to achieve a two-step sequential energy transfer (Fig. 3a) [27]. The captured photonic energy was shown to efficiently transfer to the energy acceptor to drive photocatalytic reactions. MacPhee et al*.* introduced nanofibers assembled from short peptides to guide the spatial arrangement of two individual fluorescent moieties as an artificial light harvesting antenna complex (Fig. 3b) [23] which facilitated efficient light harvesting and energy transfer thus enabling broad applications for white-light luminance [24] and photocatalysis [27].

An additional property of the artificial excitonic complex hinges upon the strongly coupled dye aggregate system [4]. A variety of self-assembled dye aggregates have been explored, such as light-harvesting nanotubes formed by amphophilic cyanine dyes [28], supramolecular assemblies from zinc-chlorins [29], tubular porphyrin dye aggregates [30], self-assembled nanofibers [31], supramolecular polymers [32], among many others, each of which demonstrated the long-range exciton motion capabilities for efficient energy transfer. In one study, Vanden Bout et al*.* synthesized and characterized double-walled tubular J-aggregates assembled by amphiphilic cyanine dyes (Fig. 3c) [28]. These dye aggregate nanotubes exhibit uniform structures with micron-scale exciton motion capabilities at room temperature [33]. Hildner et al*.* showed that self-assembled nanofibers by triarylamine derivatives, form closely stacked H-aggregates that can efficiently transport singlet excitons over more than 4 µm (Fig. 3d) [31]. In addition, self-assembled fibers from conjugated polymers [34] have also been demonstrated to conduct > 200 nm exciton transport, and such strongly coupled excitonic systems have been further integrated with energy donors or acceptors [35–38] and used for solar cells [39].

## Utilization of dye aggregates for biomedical applications

The unique optical properties of densely packed multi-chromophore complexes are of great interest in biomedical applications, especially with dye aggregates where the absorption and fluorescence emission spectra are in the near-infrared (NIR) range from 750 to 1700 nm. NIR light is advantageous because it can penetrate tissues 1–20 mm [40], and these photoactive materials have been frequently used in fundamental studies and clinical applications [41–43]. The NIR dye aggregates assembled from diverse organic pigments, including cyanine dyes, porphyrin derivatives, boron dipyrromethene (BODIPY), squaraine dyes, etc. have been employed as powerful agents for effective optical biosensing, in vivo imaging, and cancer therapy [44].

### Biosensing

**Fig. 4:**
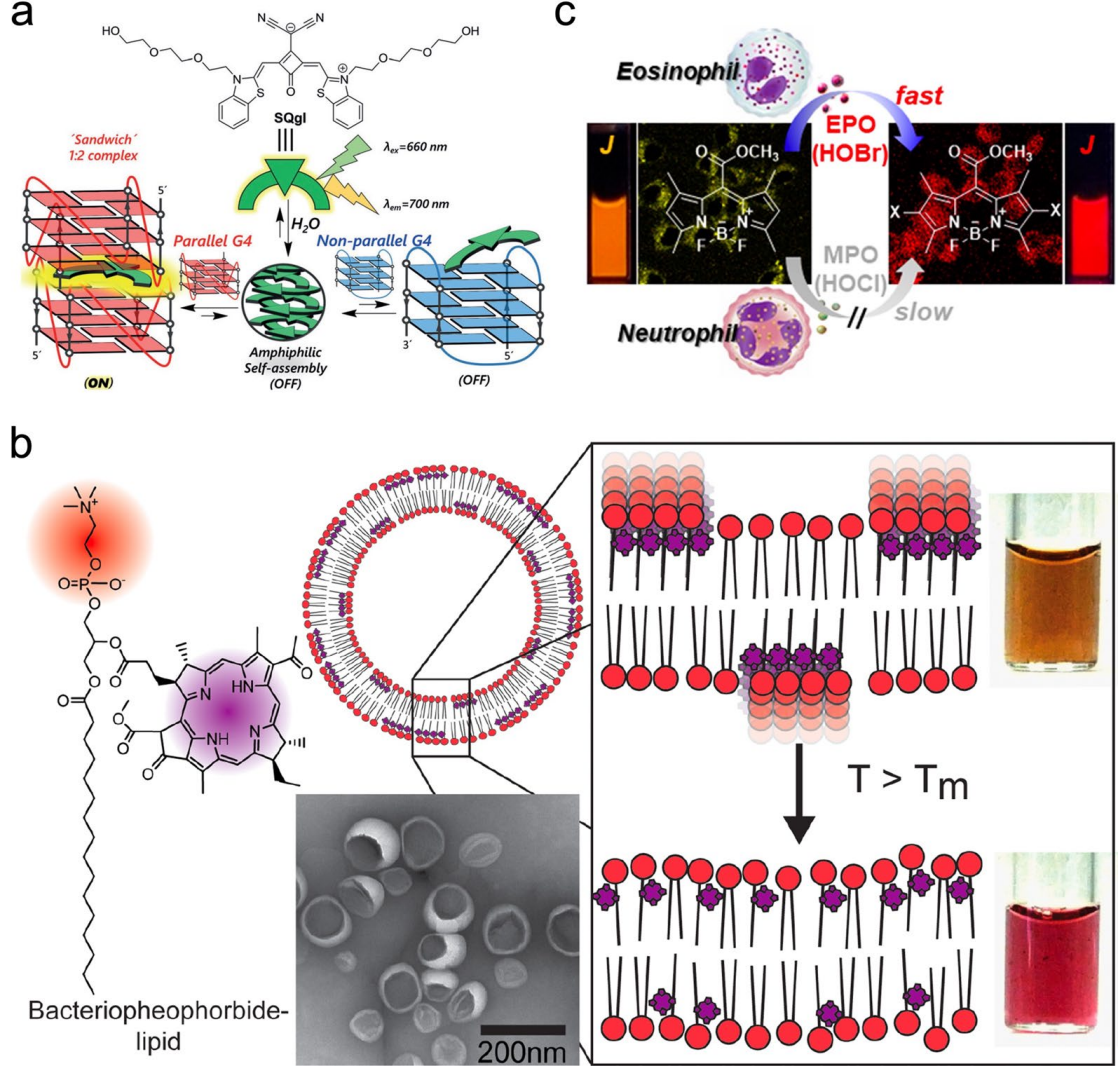
Dye aggregates for biosensing. **a** SQgl dye as a light-up probe to selectively detect parallel G4; **b** A temperature-responsive nanoswitch based on the lipid vesicle embedded with BChl dyes as a probe for in vivo temperature sensing; **c** BODIPY chromophores can be used to specifically detect the activity of eosinophil peroxidase (EPO). Reprinted with permission from: **a** Ref. [49], copyright (2017) Wiley; **b** Ref. [51], copyright (2014) American Chemical Society; **c** Ref. [52], copyright (2018) American Chemical Society

A common approach to designing an optical biosensor is the development of an efficient fluorescence probe that can selectively bind to target species [45]. The dramatic changes in spectral characteristics of the organic pigments from the monomeric to the aggregated form provide the basis for effective biosensing where the dye specifically interacts with the target molecules and convert from a fluorescence-quenched state to a fluorescence-active state to achieve fluorescence enhancement. These dye-aggregate probes have been investigated to selectively detect small molecules [46, 47], proteins [48], DNA [49], and glycosaminoglycans [50]. One example demonstrated that an amphiphilic dicyanovinyl squaraine SQgl [49] could serve as a highly selective probe to detect G-quadruplexes (G4) in the parallel configuration (Fig. 4a). In this work, it was shown that the SQgl could specifically bind with parallel G4s to form a sandwich-like 1:2 complex, which resulted in a 20,000-fold fluorescence enhancement due to the switching from a non-emissive aggregated form, to a highly emissive dye-DNA complex.

Temperature change can also cause the switching of molecular arrangement of dye molecules which consequently alter the spectral properties. In 2014, a nanoswitch based on a thermo-responsive lipid vesicle embedded with BChl dyes was introduced as a probe for in vivo non-invasive temperature monitoring [51]. The temperature-induced reversible switching of the fluidity of lipid bilayers led to the corresponding changes in spatial arrangements of the embedded BChl dye (Fig. 4b) where the BChl molecules presented a J-type stacking and red-shifted absorbance at low temperatures. Once the temperature exceeds a certain threshold that can be tuned by modification of the lipid species, the increased mobility of BChl in the membrane lead to the disruption of dye aggregates showing a significantly decreased absorbance and photoacoustic signal.

Further, the highly emissive dye aggregate system can be used as a probe to monitor enzyme activity. For example, a BODIPY chromophore has been reported to selectively detect eosinophil peroxidase (EPO) that generates HOBr from the myeloperoxidase (MPO) related to HOCl generation [52]. The chromophore shows 1200-fold higher kinetic selectivity with HOBr than that of HOCl, and the dibrominated product can self-assemble into J-aggregates exhibiting strong fluoresce emission (Fig. 4c). The fast response (time < 2 s) and highly sensitive EPO probes have been used for live cell imaging and blood sample testing.

### Bioimaging

Fluorescence imaging and photoacoustic (PA) imaging are effective tools for fundamental and clinical biomedical applications because they provide real-time wide-field images with high spatial resolution down to ~ 100 μm using low-toxic organic chromophores [42]. High quality in vivo bioimaging requires imaging agents that show strong signals, good photostability, and deep tissue penetration in pursuit of high signal-to-background ratio (SBR) and spatial resolution [53]. Leveraging the excellent optical properties of strongly coupled multi-chromophore complexes, especially J-type dye aggregates in the NIR range which serve as attractive agents for both fluorescence and PA imaging. The fluorescence imaging relays on the dye aggregates present strong fluorescence emission, while the PA imaging agents serve as non-emissive multi-pigment complexes that convert the absorbed photon energy into heat via vibration relaxation, which result in the formation of sound waves as PA signals that are detectable for ultrasound transduction [54]. In general, J-aggregates show strong, red-shifted absorption, making them ideal materials to design novel imaging agents that are active in the NIR range for deep tissue penetration. The significantly enhanced fluorescence emission spectroscopic features from J-aggregates improve the SBR for fluorescence imaging and are potentially beneficial for multiplexing thus providing the ability to avoid spectral overlaps. In contrast, the increased absorption extinction coefficient of J-aggregates compared to the monomeric species and the quenched emission in certain cases, make some J-aggregates good candidates for effective PA imaging.

**Fig. 5:**
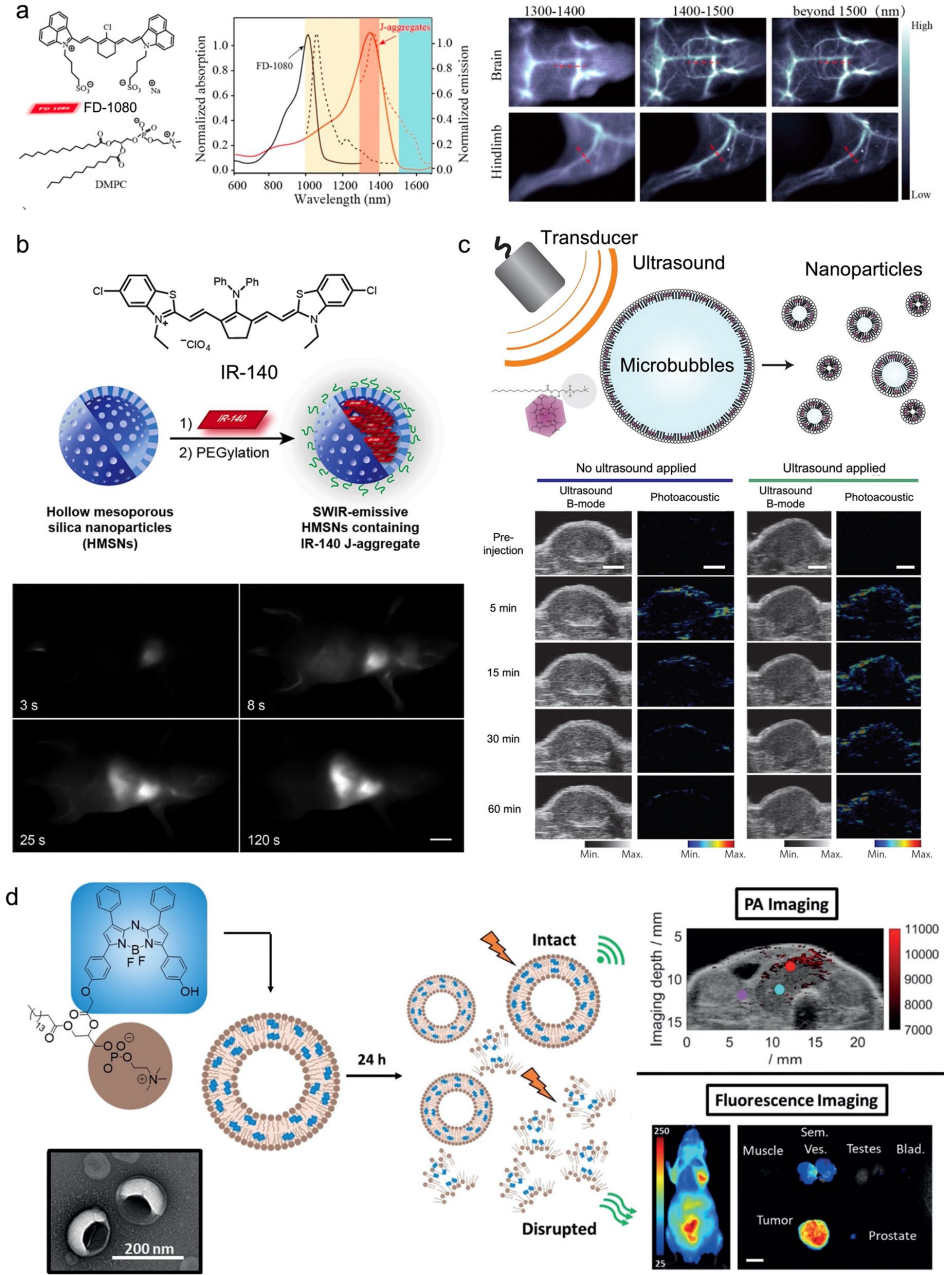
Dye aggregates for effective bioimaging. **a** Self-assembled J-aggregates from cyanine dye FD-1080 for effective bioimaging; **b** the IR-140 J-aggregates encapsulated by porous silica nanoparticles showing enhanced in vivo stability; **c** porphyrin-derivates have been embedded into the lipid assemblies to form dye aggregates serving as PA agents. The ultrasound-induced transformation from microbubbles to nanoparticles shows prolonged PA signals; **d** aza-BODIPY-lipid conjugates can assemble to BODIPYsome for dual-modal fluorescence and PA imaging. Reprinted with permission from: **a** Ref. [61], copyright (2019) American Chemical Society; **b** Ref. [65], copyright (2019) American Chemical Society; **c** Ref. [70], copyright (2015) Springer Nature; **d** Ref. [72], copyright (2019) Wiley

A series of J-aggregates that absorb and emit NIR light, including cyanine dyes [55], porphyrin derivatives [56], BODIPY [57], and squaraine dyes [58], have been reported as powerful tools to achieve effective in vivo fluorescence imaging and accomplish complex practical tasks, such as imaging‐guided osteosynthesis [59] and tracing dynamic collateral circulation processes [60]. Recently, a novel cyanine dye FD-1080 [61] was reported by Zhang et al*.* that forms J-aggregates via co-assembly with 1,2-dimyristoyl-*sn*-glycero-3-phosphocholine (DMPC) showing both absorption and emission wavelengths over 1300 nm (Fig. 5a). The great spatial resolution and high SBR of the J-aggregate imaging in the second NIR window (1000–1700 nm) enable in situ dynamic monitoring for the blood width of the carotid artery expanding from 370 to 680 μm after treatment of the hypertensive rate with hypotensor.

The challenges in the application of dye aggregates for bioimaging are their structural and spectral stability and long-term blood circulation. For example, indocyanine green (ICG), an FDA-approved clinical NIR imaging agent, has been reported to bind with serum proteins resulting in the switching between aggregated and monomeric forms which consequently change the spectral features [62]. To improve the stability and circulation of dye aggregates in vivo, synthetic scaffolds, such as polymers, inorganic nanoparticles, and lipid vesicles, have been used to co-assemble [63] or encapsulate [64] the densely packed dye aggregates. Sletten et al. have reported the use of hollow mesoporous silica nanoparticles to trap the J-aggregates formed by the NIR chromophore IR-140 [65], where the dye aggregates were encapsulated by nanoparticles which showed enhanced stability and have been used to achieve efficient in vivo imaging (Fig. 5b).

Effective PA imaging predominantly depends on the dye aggregates showing quenched fluorescence emission. PA imaging agents are prepared via templating or encapsulating those densely stacked dye aggregates using the self-assembled lipid [66], lipoprotein [67], and polymer [68] nanocarriers, which can facilitate the lifetime of blood circulation and accumulation of the dye at the tumor site. Inspired by natural light-harvesting systems, Zheng et al. developed a series of porphyrin dye aggregates assisted by lipid assemblies for photoacoustic applications [69]. Self-assembled lipid structures in multiple sizes ranging from 100 nm [51, 56] to 10 µm [70] direct the formation of dye aggregates with ordered spatial arrangements assembled from diverse porphyrin derivatives [71]. Those dye aggregates embedded in the lipid structures exhibited a bathochromic shift of absorption, indicating a J-type “head-to-tail” molecular arrangement, resulting in fluorescence quenching, and strong PA signal enhancement. One example introduced the BChl molecules into the microbubbles (pMBs, a contrast agent for ultrasound imaging) with gas encapsulated (Fig. 5c) for multimodal imaging (ultrasound, fluorescence, and PA) [70]. The treatment of ultrasound can induce the conversion of pMBs to nanoparticles (pNPs) at the tumor site while PA properties are retained for a prolonged retention time. This in situ conversion quickly delivers the pNPs into target sites in minutes which indicates potential applications in drug delivery. In addition, a variety of biocompatible PA agents based on BODIPY [72], ICG [73], squaraine dyes [66], etc. have also been reported for PA imaging in tumor model mice. The nanocarriers can be further conjugated with antibodies for specific tumor targeting, which boosts the efficacy and quantification of PA imaging, making it a promising candidate for future clinical applications [74].

Furthermore, some J-aggregate-based imaging agents have been shown as dual-modular probes for both fluorescence and PA imaging [66, 75, 76]. Zheng et al*.* showed that the aza-BODIPY-lipid conjugate co-assembled with phospholipids to form the vesicle BODIPYsome [72] which presented the red-shifted absorption peak and > 96% fluorescence quenching (Fig. 5d). The BODIPYsome presents good serum stability and strong PA signals in the NIR range, allowing it to be successfully used as a PA agent for in vivo tumor imaging. Notably, after 6 and 24 h post-injection, a strong fluorescence signal in the tumor site was observed, because BODIPYsomes accumulating in the tumor tissue were disrupted to recover the fluorescence emission, making the assembled BODIPY-based nanostructures attractive dual-modular imaging agents.

### Cancer therapy

Phototherapy, including photothermal therapy (PTT) and photodynamic therapy (PDT), has been demonstrated as an effective treatment of cancer through the conversion of photon energy to produce hyperthermia to generate cytotoxic species to effectively eliminate tumor cells [77]. Non-emissive dye aggregates, especially J-type aggregates, can serve as good therapeutic materials for cancer phototherapy due to their excellent spectral properties including narrow and significantly red-shifted absorption spectra in the NIR window, and increased absorption extinction coefficient compared to the monomeric form.

**Fig. 6:**
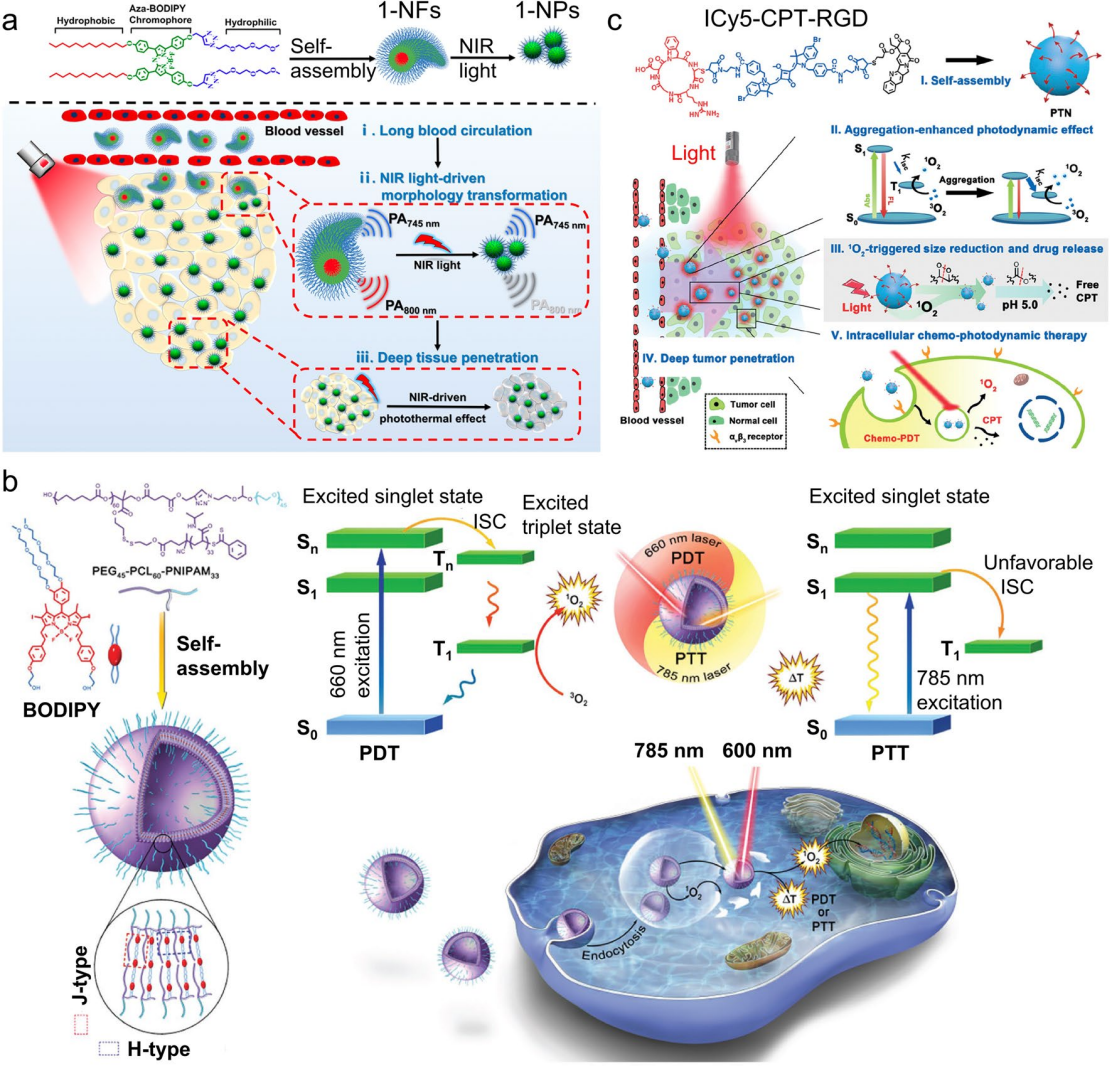
Dye aggregates for cancer phototherapy. **a** aza-BODIPY nanoaggregates show the transformable morphology from fibers (1-NFs) for long blood circulation times to particles (1-NPs) for deep tumor penetration; **b** BODIPY dye aggregates nanoparticles show a wavelength-dependent phototherapy, i.e., effective PDT using 660 nm excitation and effective PTT using 785 nm excitation. **c** Self-assembled Icy5 dye conjugates for combined PDT and chemotherapy. Reprinted with permission from: **a** Ref. [84], copyright (2020) American Chemical Society; **b** Ref. [92], copyright (2017) Wiley; **c** Ref. [98], copyright (2018) Wiley

The high photothermal conversion efficiency and photo-stability due to enhanced non-radiative decay pathways make dye aggregates ideal candidates for effective photothermal therapies [78]. The J- or H-aggregates assembled from ICG [79], hyperbranched porphyrin [80], BODIPY [81, 82], squaraine dye [58], tetramethylbenzidine [83], etc. have been demonstrated as good PTT agents with efficient photothermal conversion, low toxicity, and efficient antitumor performance. Moreover, the rational design in the morphology of assembled dye aggregates was carried out to further improve the circulation, accumulation, and penetration of the photothermal agents in vivo. Wang et al*.* introduced morphologically transformable nanoaggregates assembled by aza-BODIPY dyes to achieve long blood circulation time and deep tumor penetration [84] (Fig. 6a). The amphiphilic dye monomers first assemble into nanofibers (1-NFs) which then can transform to spherical nanoparticles (1-NPs) triggered by NIR light irradiation. The 1-NFs showed a 7.6-fold longer circulation time in the blood compared to that of 1-NPs. The fiber-to-particle transformation can be performed in vivo after accumulation at the tumor site. The 1-NPs showed high photothermal conversion efficiency, good thermal stability, deep tissue penetration, and effective tumor inhibition.

Some dye aggregates [85, 86] have been demonstrated to show effective photodynamic therapy under NIR irradiation, which relays across the intersystem to form a triplet state for the generation of reactive oxygen species (ROS) that leads to cytotoxic effects on tumor cells [87]. In some cases, the photodynamic J-aggregate agents also show a good photothermal conversion for both enhanced PDT and PTT [88–90] where their properties can also be tuned by the irradiation wavelength [91]. For example, a BODIPY-based dye aggregate vesicle [92] presents either strong PDT performance upon excitation at 680 nm or effective PTT under 785 nm irradiation, allowing for a wavelength-dependent cancer phototherapy (Fig. 6b).

The self-assembled dye aggregate systems, alone [80, 90, 93] or combined with other imaging agents [94–96], have been employed for imaging-guided phototherapy. Liu et al. has developed a dye aggregate complex with iron oxide nanoparticles [95] that are contrasting agents for magnetic resonance (MR) imaging. The complex showed effective photothermal therapy, and the MR imaging revealed substantial tumor accumulation. Furthermore, the dye-aggregate-based phototherapy could be combined with a chemodrug [97, 98] for synergistic cancer therapy. Yin et al*.* reported a multi-functional nanodrug assembled from pentamethine indocyanine (ICy5) dye [98] (Fig. 6c). The densely packed ICy5 dye aggregates exhibit appropriate photodynamic properties for effective PDT. Further, the singlet oxygen generated by PDT results in the reduction of the size of nanodrug, which triggers the release of encapsulated chemodrugs for the chemo-PDT combined therapy. Additionally, stimuli-responsive dye aggregated systems have also been explored in pursuit of smart and precise cancer therapy [99, 100]. In 2016, Zheng et al*.* introduced photothermal enhancing auto-regulated liposomes (PEARLs) to achieve deep and homogeneous photothermal therapies [101]. The absorption properties of J-type porphyrin aggregates embedded in liposomes can be reversibly tuned by temperature change during PTT. The PTT-induced hyperthermia in the shallow layer of tissue leads to better transmission of irradiatied light, and consequently increased light penetration for heating of deep tissue.

## DNA nanotechnology

DNA is a well known biomolecule encoding the genetic information that guides the biological activities of life. In the past four decades, DNA has emerged as a programmable material devoid of biological function, to construct nanostructures with a ‘bottom-up’ approach [102–104] which utilizes intrinsic molecular information of building blocks to direct their autonomous assembly for higher-order architectures.

DNA is a programmable polymer containing precise molecular recognition due to highly specific Watson–Crick base-paring. Naturally occurring DNA is composed of two single-stranded DNA (ssDNA) strands, and each ssDNA oligonucleotide is formed by covalently linked nucleotides as repeating units containing a phosphate group, a deoxyribose, and a nucleobase. DNA is comprised of four nucleobases: adenine (A), thymine (T), cytosine (C), and guanine (G). The specific and predictable Watson–Crick pairing between nucleobases, where A pairs with T and C pairs with G, ensures the precise binding between ssDNA strands and can be introduced to program the interaction between each strand for both its well-defined primary and secondary structure. B-form DNA exhibits a right-handed double helix structure with a diameter of 2 nm, a helical pitch of 3.4 nm, and 10.5 base pairs per turn which offer opportunities for the precise design and organization of DNA-based nanostructures. In addition, the well-established solid-phase synthesis of DNA with various chemical modification, enables the large-scale and low-cost usage of synthetic DNA. This can also be coupled with a variety of enzymatic manipulations that can be used to tune and modify DNA oligos. The development of DNA nanotechnology allows for the design and construction of DNA tile-based and origami assemblies.

### DNA tile assembly

**Fig. 7:**
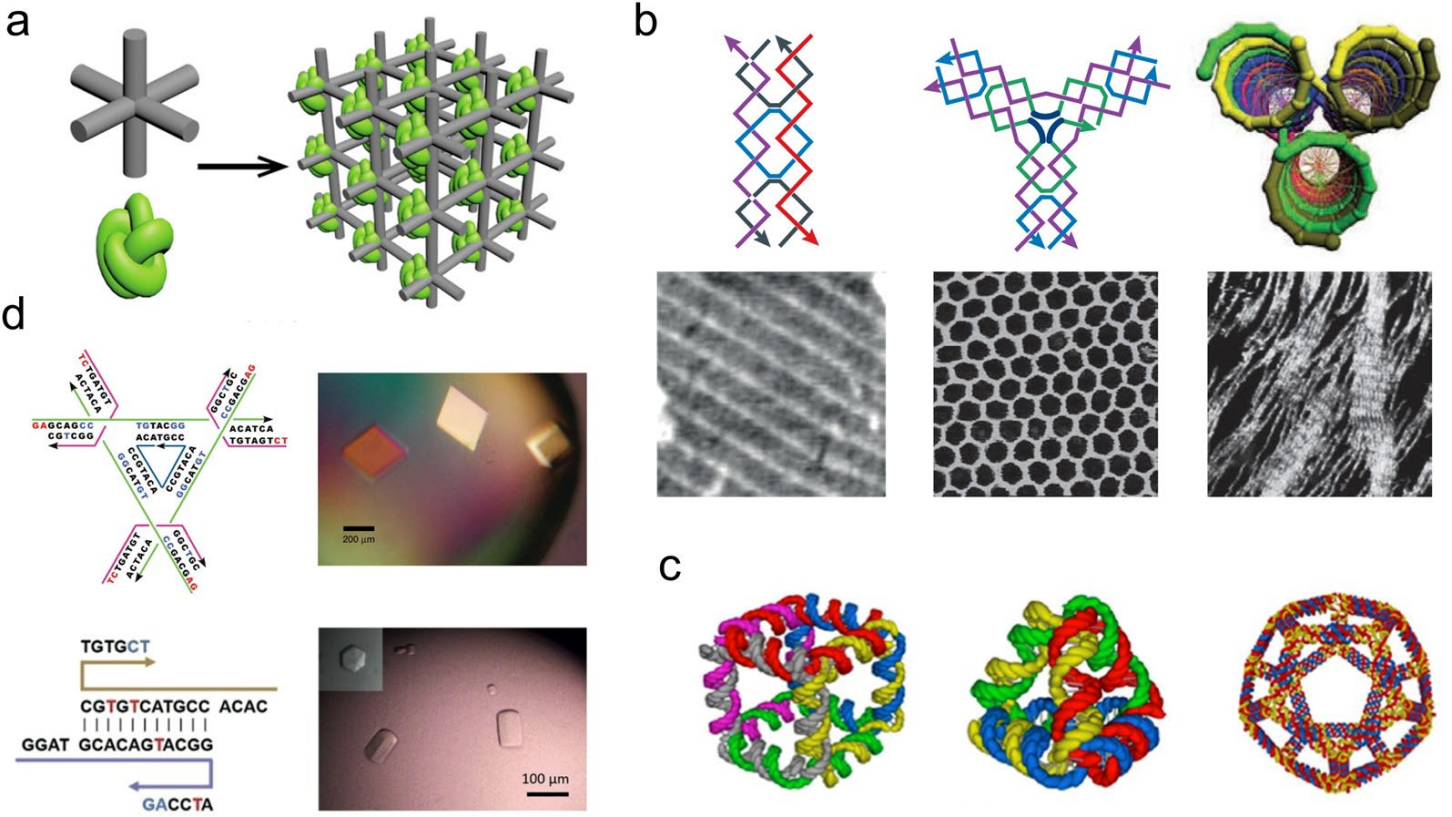
DNA nanostructures based on tile assembly. **a** Seeman’s proposal about utilization of branched DNA motifs to create 3D scaffold for protein crystallization [105]. **b** Examples of 2D arrays assembled by DX tile (left) [108], three-point-star tile (middle) [115], and three-helix bundle (right) [112]. **c** Examples of DNA assembled cube (left) [120], tetrahedron (middle) [121], and dodecahedron (right) [122]. **d** 3D DNA crystals formed by tensegrity triangle (top) [123], and six-symmetry motif (bottom) [127]. Reprinted with permission from: **a** Ref. [142], copyright (2017) American Chemical Society; **b** Ref. [103], copyright (2017) Springer Nature; **c** Ref. [104], copyright (2014) American Chemical Society; **d** top: Ref. [123], copyright (2009) Springer Nature; bottom: Ref. [127], copyright (2018) Wiley

In 1982, Nadrian Seeman, the pioneer and founder of structural DNA nanotechnology, proposed the utilization of branched DNA motifs to construct three-dimensional (3D) lattices via hybridization of sticky ends (ssDNA overhangs) to orient proteins for crystallization [105]. (Fig. 7a) Biologically occurring branched DNA structures like the Holliday junction (HJ), show intrinsic limitations due to their geometric flexibility and sequential symmetry. To overcome those limitations, Seeman reported the immobile four-armed HJ with unique asymmetric sequences for each arm [106]. Double-crossover (DX) structures were further developed where two DNA helices are joined by two crossovers and showed improved rigidity of adjacent duplexes that are tethered by HJ crossover points [107]. These DNA “tiles” can be used as the building units to construct higher-order architectures. In 1998, the first two-dimensional (2D) periodic DNA crystal was reported by the usage of DX tiles [108].

This construct was further expanded to a triple-crossover [109], four-helix [110], eight-helix, and twelve-helix tile [111], and three-helix [112] and six-helix bundles [113]. Additionally, a variety of DNA tiles with various morphologies, including the 4 × 4 [114], three-point-star [115], and six-point-star [116], were developed utilizing a central strand to connect multiple arms. Those basic units were capable of forming DNA nanotubes and a 2D lattice with periodic patterns [117] (Fig. 7b). Archimedean tiling [118] and DNA 2D quasi-crystal [119] were also achieved by employing multiple types of tiles with rational junction designs and matching rules. A variety of 3D polyhedra, such as cubes [120], tetrahedra [121], dodecahedrons, buckyballs [122], amongst a host of others, were also constructed based on multi-arm junctions and DX components (Fig. 7c). The 3D DNA crystals proposed in 1982 have been reported using a rationally designed tensegrity triangle [123], various 4 × N motifs [124–126], a six-fold symmetric motif [127], and a layer-crossed motif [128] (Fig. 7d). ssDNAs have also been used as self-assembly units, such as single-stranded tiles (SSTs), to generate arbitrary 2D [129] and 3D shapes [130].

### DNA origami assembly

**Fig. 8.**
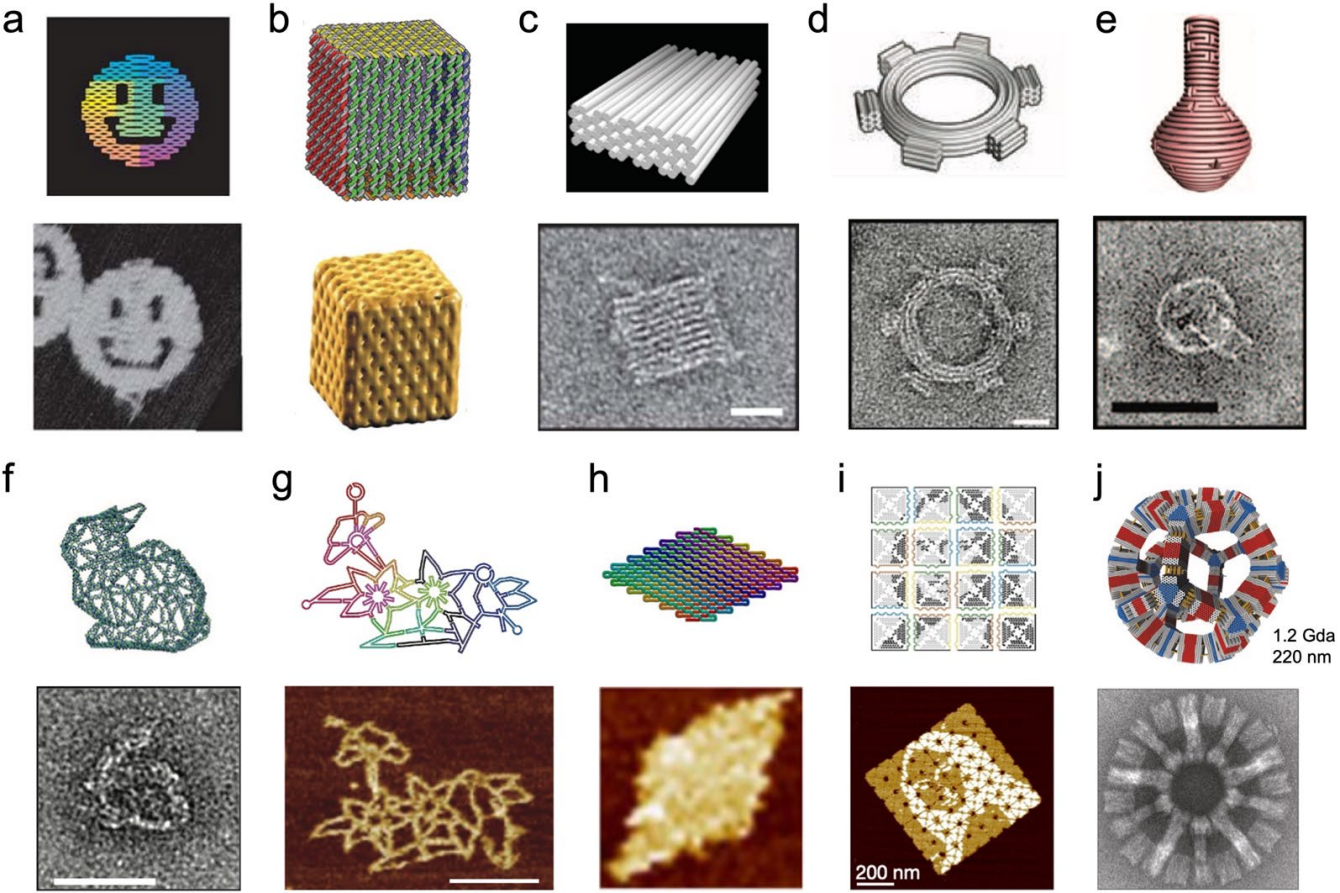
2D and 3D DNA origami. **a** 2D smiley face [131], **b** 3D cube, [132], **c** 3D multi-layer structure with honeycomb lattice [134], **d** 3D DNA gear [136], **e** 3D DNA vase [137], **f** 3D DNA mesh [139], **g** 2D wireframe DNA origami [138], **h** single-stranded DNA origami [140], scaled-up DNA origami with **i** complicated patterns[143] and **j** gigadoltan scale [144]. Reprinted with permission from: **a** Ref. [131], copyright (2006) Springer Nature; **b** Ref. [132] copyright (2009) Springer Nature; **c** Ref. [134], copyright (2009) Springer Nature; **d** Ref. [136], copyright (2009) AAAS; **e** Ref. [137], copyright (2011) AAAS; **f** Ref. [139], copyright (2015) Springer Nature; **g** Ref. [138], copyright (2015) Springer Nature; **h** Ref. [140] copyright (2017) AAAS; **i** Ref. [143], copyright (2017) Springer Nature; **j** Ref. [144] copyright (2017) Springer Nature

The DNA origami design strategy was introduced by Paul Rothemund in 2006 [131]. A long viral ssDNA “scaffold” strand with a length of several thousands of nucleotides was folded to arbitrary 2D nanostructures with the DX-based structural design which was assisted by hundreds of short ssDNAs “staple” oligonucleotides. It has been shown that planar 2D DNA origami can be further folded to 3D objects via “cutting” the 2D sheet as several connected sub-units, such as squares and triangles, as faces of cubes [132] and tetrahedra [133], and subsequently jointed these motifs with designed shapes to specified 3D architectures. To overcome the flexibility of single-layered origami, the strategy utilized to design a compact 3D DNA origami structure allows for the stacking of single-layer designs to create multiple layers with honeycomb [134] and square lattices [135]. Furthermore, more complex structural features, including twist [136] and curvature [137], have been implemented into 3D DNA origami designs (Fig. 8).

DNA origami has also developed methods where the component DNA helices can be aligned beyond the DX context. The wireframe DNA origami was reported to construct complex 2D structures and arbitrary 3D meshes using DX tiles [138] or double stranded DNA (dsDNA) [139] at each edge. ssDNA origami was also created by self-folding of a ssDNA strand that contained a partial complement to the desired shape via parallel crossover cohesion [140]. Owing to the high degree of programmability and intrinsic spatial addressability of DNA origami, 2D and 3D objects at the nanoscale have been utilized as versatile templates to engineer functional components for broad applications that include fundamental biophysical studies to complex nanodevices and nanorobots [141, 142]. Self-assembled DNA origami can further be scaled up to generate higher-order structures via base-pairing or shape complementarity to achieve sophisticated patterns [143], gigadalton-scale objects [144], and macroscopic 3D crystals [145].

### Dynamic DNA technology

**Fig. 9:**
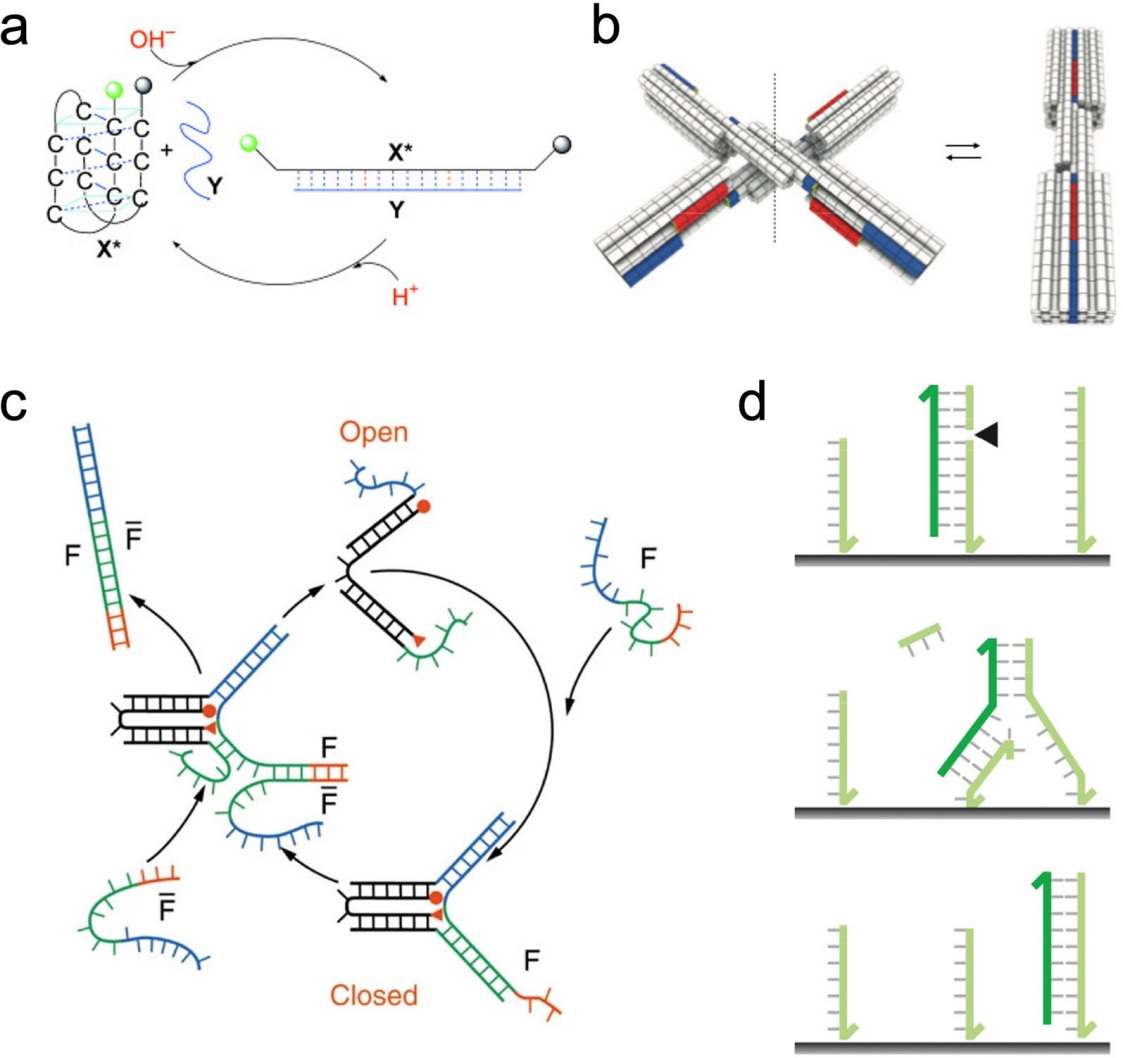
Dynamic DNA devices. **a** DNA nanomotor fueled by proton [151]. **b** Reconfigurable 3D DNA structures [154]. **c** DNA tweeter fueled by DNA strand displacement reaction [148]. **d** A unidirectional DNA walker [150]. Reprinted with permission from: **a** Ref. [151], copyright (2003) Wiley; **b** Ref. [154] copyright (2015) AAAS; **c** Ref. [148], copyright (2009) Springer Nature; **d** Ref. [146], copyright (2007) Springer Nature

Another dynamic aspect of DNA nanotechnology is the ability to generate nanodevices with non-equilibrium dynamics based upon the programmability of DNA molecules [146, 147]. A variety of DNA nanodevices, such as tweezers [148], actuators [149], walkers [150], and circuits, have been developed to bring out switching, reconfiguration, motion, computation, and more (Fig. 9).

Mechanisms to power dynamic DNA nanostructures can be categorized as non-DNA and DNA-based types. In the non-DNA triggered system, the conformation of DNA structure or interaction of the DNA helix can be tuned by the external stimulus including pH [151], light [152], ionic strength [153], small molecules, temperature [154], enzyme, electronic field [155] among others, which can be used to drive dynamic DNA devices. DNA-based dynamic systems primarily relies on strand displacement. Strand displacement is used to displace the pre-hybridized strands from a multi-stranded complex by employing a partially or fully complementary strand. The reaction has been used to construct reconfigurable DNA structures [156], stepped walkers [157], logic circuits [158], among many others. The complexity of DNA nanodevices can be further expanded for cargo sorting [159], targeted drug delivery [160], and reconfigurable nanophotonic systems [161].

## Photonic systems directed by structural DNA templates

The artificial photonic systems for efficient light-harvesting and energy transfer require the precise control of the position, orientation, dynamics, and environment of photonic components, including chromophores and nanoparticles. Although numerous examples of biomimetic photonic systems have been developed, it is still challenging to rationally design and construct photonic architectures that can have well-controlled sizes and geometries as well as the spatial arrangement of photoactive components. Structural DNA nanotechnology provides a feasible approach to address these long-standing challenges that utilize DNA assemblies as templates to arrange photoactive species in a programmable manner.

Programmable DNA templates show various sizes ranging from a few nanometers to micron-scale and extendable dimensions from 1 to 3D. The intrinsic addressability of self-assembled DNA nanostructures offer nanometer precision to organize photonic components covalently or non-covalently with designed distance, orientation, and stoichiometry. Those advantages empower DNA-directed photonic systems to serve as versatile templates for both fundamental studies to reveal poorly understood phenomena, such as exciton motion [162], multi-chromophore energy transfer [163], etc. and functional applications in excitonic switches [164, 165] and information encryption [166]. In addition, the dynamic DNA structures can also be integrated into DNA templates to design and construct responsive and reconfigurable photonic complexes for biomedical applications.

DNA nanostructure based photonic systems fall into two broad categories: (1) to organize chromophores and quantum dots on the DNA-based platforms to mediate the cascaded excitation transport based on Förster Resonance Energy Transfer (FRET) mechanism; and (2) the employment of DNA nanostructures as templates for assembly of strongly coupled dye complexes with a densely packed molecular arrangement, or for routing of the conjugated polymer to direct excitation energy transfer beyond the FRET regime of a few nanometers.

### Organization of weakly coupled chromophores

**Fig. 10:**
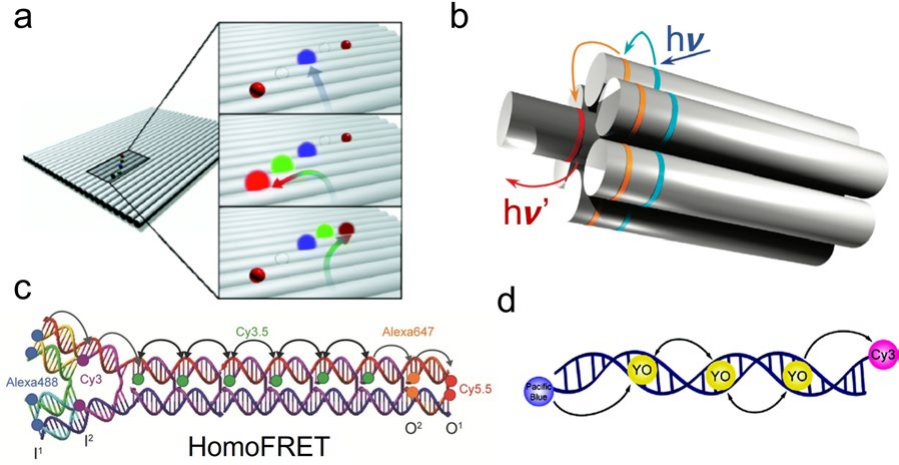
DNA templates for precise control of weakly coupled chromophores. **a** Using DNA origami to precisely organize multiple dyes [173]. **b** An artificial light-harvesting antenna organized by DNA seven-helix-bundle [26]. **c** A cluster of Cy3.5 dyes to conduct homo-FRET for long-range energy transfer [178]. **d** A photonic wire based on DNA duplex [182]. Reprinted with permission from: **a** Ref. [173], copyright (2011) American Chemical Society; **b** Ref. [26], copyright (2011) American Chemical Society; **c** Ref. [178], copyright (2016) Wiley; **d** Ref. [182], copyright (2008) American Chemical Society

The integration of chromophores to DNA structures dominantly relies on the covalent modification of specific dye molecules to DNA strands at 5’- or 3’-ends, or internally, via phosphoramidite chemistry or post-synthesis conjugation [167, 168]. The dye-DNA conjugates were incorporated into the self-assembled DNA platforms, including dsDNA [168–171], DNA tiles [26, 172], DNA bricks [163], and DNA origami [173–175], for the precise spatial arrangement and stoichiometric control of multiple chromophores with rationally designed spectral features. For example, DNA origami can be used to arrange the spatial patterns of fluorophores for a cascaded FRET signal along controlled pathways (Fig. 10a). Those DNA-based weakly coupled multi-chromophore systems conducting the cascaded energy transfer were introduced for molecular photonics and artificial light-harvesting. For instance, a DNA-directed light-harvesting antenna has been synthesized by using the self-assembled seven-helix DNA bundle to organize pyrene molecules, Cy3 dyes, and Alexa Fluor 647 molecules to form circle arrays as primary donors, second donors, and acceptors, respectively. These artificial antenna complexes enable an efficient, stepwise funneling of the excitation energy (Fig. 10b). To facilitate the long-range energy transfer across a spatial distance of > 10 nm, a cluster of identical dyes for homogenous FRET (homo-FRET) was developed to minimize the energy loss and the distance for the cascaded excitation transport up to 30 nm have been reported [176–178] (Fig.10c).

Another approach for the construction of the DNA-dye system is the implementation of a dye that can be intercalated into the DNA helix [179–181]. Specifically the YO-PRO-1 dye can be intercalated to dsDNA that delivers energy from donor to acceptor with improved light-harvesting performance [182] (Fig. 10d). Quantum dots (QDs) have also been incorporated into DNA nanostructures as light-harvesting components due to their exceptional optical properties, such as narrow emission band, high quantum yield, and stability against photobleaching. A DNA photonic wire can be conjugated onto the surface of QDs, which serve as an energy donor to collect photons and transfer the excitation to downstream small molecular fluorophores [183, 184]. Moreover, the chromophore-DNA complex can combine with other functional components, that include photosynthetic reaction center proteins for energy conversion and artificial photosynthesis [185].

### Assembly of strongly coupled excitonic complexes

**Fig. 11:**
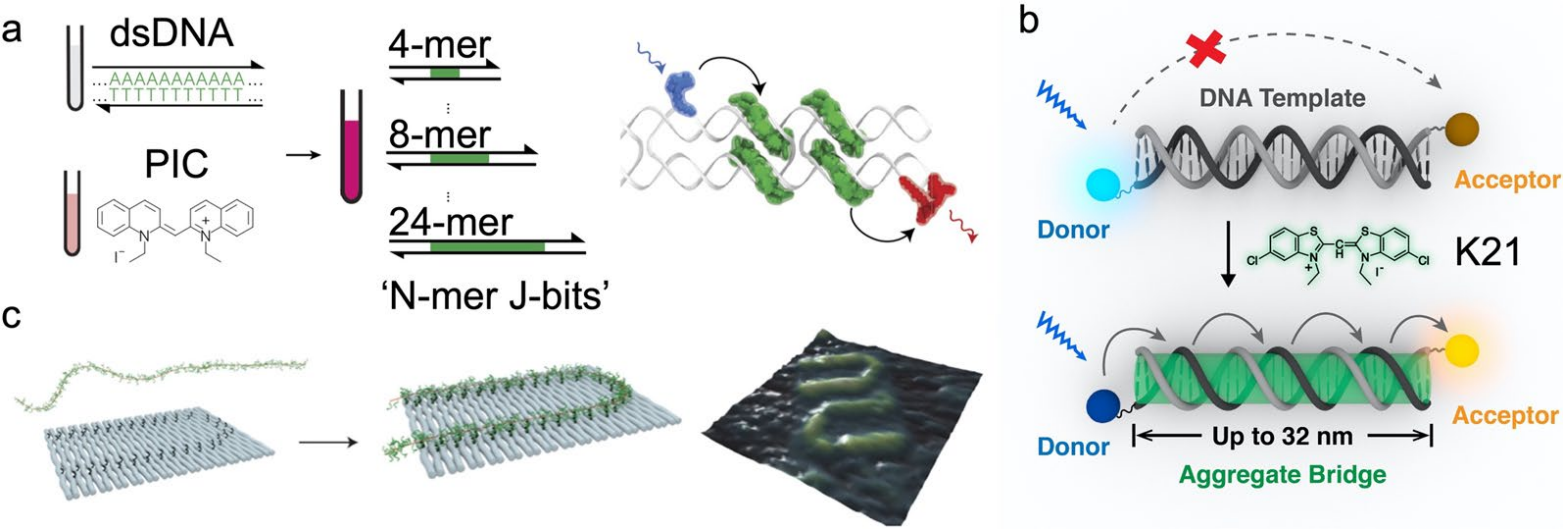
Strongly coupled excitonic systems organized by DNA templates. **a** DNA-templated PIC dye aggregates were used to mediate energy transfer on DX tile. **b** DNA duplex has been used as a template to organize cyanine dye K21 to form dye aggregates, serving as a bridge to conduct efficient donor-to-acceptor energy transfer over up to 30 nm. **c** Using DNA origami to route the conjugated polymer. Reprinted with permission from: **a** Ref. [189], copyright (2018) Springer Nature; **b** Ref. [190], copyright (2019) American Chemical Society; **c** Ref. [199], copyright (2015) Springer Nature

The self-assembled dye aggregate complex presents strongly coupled excitonic features and is capable of mediating micron-scale exciton propagation. However, those densely packed dye complexes lack precise geometry control and site-specific functionalization. To overcome those limitations, DNA structures were utilized as templates to arrange dye molecules into strongly coupled dye aggregates. A variety of dye molecules have been reported to form J- or H-aggregates via non-covalent binding onto the minor groove of dsDNA in close proximity that have been validated by their spectral features [186]. Recently, the pseudoisocyanine (PIC) dye has been demonstrated to selectively form J-like dye aggregates on poly(A)/ploy(T) dsDNA as ‘J-bits’ [187, 188], which can be engineered by DX tiles to mediate donor to acceptor energy transfer [189] (Fig. 11a). In addition, a benzothiazole cyanine dye, K21, has been demonstrated to form J-like aggregates templated on a DNA duplex which exhibit strongly coupled excitonic features and sequence independence [190]. The DNA-templated K21 aggregates have been used as “excitonic wires” to mediate efficient directional excitation energy transport over a distance up to 30 nm (Fig. 11b). Those DNA-directed excitonic systems pave the way for the design and synthesis of higher-order excitonic architectures with high geometric complexity and programmability.

DNA structures can also be used to organize the covalently conjugate dye molecules to form strongly coupled dye complexes with precise position and orientation control. The Cy3 and Cy5 dyes were reported to form dye complexes with strong electronic coupling, such as J-like dimer, H-like dimer, and H-like tetramer [191–197]. These systems were investigated to reveal the spectral properties, coherence, and dynamics of excitons, and offer new insight towards the design and application of DNA-templated strongly coupled dye complexes.

Conjugated polymers are also able to mediate long-range exciton transport because of their intrinsic electronic structure [198]. The combination of conjugated polymers onto addressable DNA nanostructures can control and tune the alignment of polymers for directional exciton transport. Gothelf et al. reported the ability to route phenylene vinylene [199] and fluorene[200] based polymer-DNA conjugates along defined tracks on the surface of rectangular DNA origami (Fig. 11c). The alignment of polymers can be switched between two different tracks using toehold-mediated strand displacement [201] to achieve a tunable excitation energy transport along a defined pathway. Subsequently, the conjugated polymers have been used as single-molecular photonic wires to conduct donor-to-acceptor energy transfer over 24 nm [202].

### Applications in phototheranostics

**Fig. 12:**
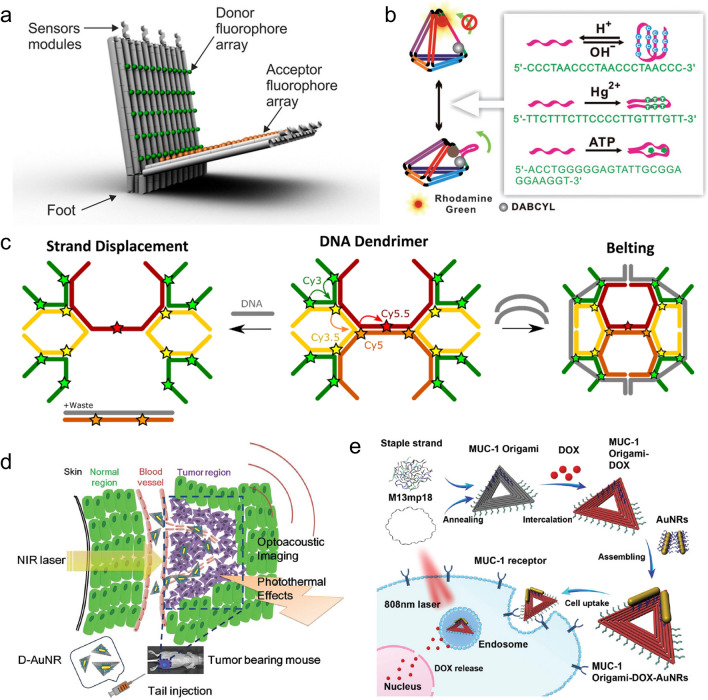
DNA-directed photonic systems for biosensing and cancer therapy. **a** A DNA origami beacon containing donor and acceptor fluorophore arrays; **b** Reconfigurable DNA tetrahedra for the construction of multiple logic gates for intracellular sensing; **c** Changing the energy transfer pathways on DNA dendrimers for applications in biosensing; **d** DNA origami hybrid with gold nanorods for enhanced in vivo PA imaging and photothermal cancer therapy; **e** DNA orgami/gold nanorods loading with DOX for synergistic PTT and chemotherapy. Reprinted with permission from: **a** Ref. [212], copyright (2018) American Chemical Society; **b** Ref. [213], copyright (2012) Wiley; **c** Ref. [216], copyright (2017) American Chemical Society; **d** Ref. [225], copyright (2016) Wiley; **e** Ref. [227], copyright (2012) Royal Society of Chemistry

DNA nanostructures empower the biomimetic photoactive systems with precise and tunable spatial arrangement, which offers more possibilities for the development of powerful tools for postdiagnosis and phototherapy. One important attribute is the implementation of the DNA-directed multi-chromophore complexes for fluorescence-based biosensing. The simplest DNA-based fluorescent biosensor is a molecular beacon (MB) [203]. The configuration of MB switches from a hairpin form to a duplex form after binding with target molecules results in the spatial separation of fluorophores and quenchers, and restore the fluorescence signal as a light-up biosensor [204]. This principle has also been applied to design biosensors by employing stimuli-responsive motifs into DNA photonic systems based on complex DNA nanostructures [205–207] for the detection of diverse targets including miRNA [208], ATP [209], pH [210], ion [211], and more. Andersen et al*.* has reported a DNA origami beacon [212] in which multi-fluorophore arrays consisting of up to 60 donor and acceptor molecules were attached. The fluorophore arrays increased the signal-to-noise ratio, and enable single device biosensing to detect target DNA sequences down to 100 pM in < 45 min (Fig. 12a). The complex DNA structures provide more flexibility to integrate multiple dynamic DNA motifs responsive to different environmental cues for constructing logic sensors. As an example, Fan et al. integrated a series of reconfigurable DNA structures, including a pH-responsive i-motif, anti-ATP aptamer, mercury-specific oligonucleotide, and hairpin structures, extending to DNA tetrahedra for the construction of AND, OR, XOR, and INH logic gates [213] (Fig. 12b). The logic sensor has been used for intracellular detection of ATP in living cells via fluorescence imaging. Compared to the simplest molecular beacon system, such photonic systems directed by complex DNA structures offer modular and robust templates that can be easily integrated into various responsive domains for multiplexed detection with high sensitivity and molecular computation to meet complex tasks.

Another strategy for using DNA-dye complexes as biosensors is to program energy transfer pathways in the cascaded FRET systems. These DNA-directed multichromophore complexes not only serve as long-range energy transfer complexes, but can also be engineered as reconfigurable excitonic logic gates [165, 214, 215] for applications in biosensing for DNA [216], pH [217], thrombin [218]. Medintz et al*.* reported a dynamic photonic network directed by the DNA dendrimer scaffold in which Cy3 is an initial donor to transfer the captured energy to a final acceptor Cy5.5 through the Cy3.5 and Cy5 as intermediate dyes [216]. The displacement or replacement of the strand labeled with Cy3.5 or Cy5 was shown to potentially interrupt the FRET paths and cause decreased energy transfer efficiency and acceptor emission (Fig. 12c).

DNA nanostructures have also been demonstrated as excellent biocompatible nanocarriers showing programmable geometries and functions for effective cancer therapy [219–221]. In the field of phototherapy, photothermal and photodynamic components can be anchored onto DNA templates for precise and effective PTT and PDT, respectively [222]. In one study, the gold nanorod (AuNR) [223], a frequently used photothermal agent, was attached to DNA origami nanostructures for promising photothermal treatment of breast cancer in mice [224] and enhanced photoacoustic imaging [225] (Fig. 12d). In addition, DNA nanostructures can load photosensitizers, such as carbazole derivative BMEPC [226], for both effective imaging and PDT for MCF-7 cells. The DNA-based PTT systems also can be combined with a chemotherapy drug, DOX, for synergistic therapy for multidrug-resistant tumors [227] (Fig. 12e).

## Summaries and perspectives

Bioinspired multi-chromophore complexes, especially the densely packed dye aggregates, show a well-controlled molecular arrangement and unique spectral characteristics. Employing DNA assemblies as structural templates provide high-level spatial precision and geometric complexity. These photonic systems have been used for efficient light-harvesting and excitation energy transport. Further, these self-assembled complexes based on organic chromophores, in many cases assisted by synthetic scaffolds, show excellent applications in biosensing, in vivo fluorescence/PA bioimaging, and cancer phototherapy.

Despite the considerable progress in the combination of DNA nanotechnology and biomimetic photonic systems for energy and biomedicine, the barriers in the development of structures and associated properties and functions of these DNA-directed multi-chromophore systems still need more effort to overcome. First, a crucial question, how precisely the position and orientation control can be achieved at the atomic level using DNA nanostructures as templates. Recent studies [228, 229] have shown that dye attachment chemistry, anchoring sites, and local microenvironment can impact the spectral properties and orientation of fluorophores. Hence, the well-chosen sites to anchor chromophores are critical for the design of complex photonic systems with efficient energy transfer performance. Second, current DNA-templated strongly coupled photonic systems are predominantly based on simple DNA assemblies, such as the fundamental helix and a higher-order DX tile. It would be an extreme accomplishment to introduce complex DNA templates with programmable geometry and extended dimension, including DNA origami, DNA array, and DNA crystal, to form higher-order photonic architectures for long-range energy transfer and large-scale light-harvesting. While many dye molecules have been reported to form strongly coupled assemblies either with or without the direction of DNA, their molecular arrangements and interactions have not been determined and their capabilities for mediating excitation energy transport have not been investigated in detail. More experimental approaches, including, but not limited to, time-resolved spectroscopy, near-field optical microscopy, X-ray crystallography, etc., and theoretical modeling are in demand for understanding their structures, and the correlation between their chemical behavior and spectral properties, which are vital for the improvement of artificial photonic systems. Third, the stability of DNA-templated excitonic complexes, especially in long-term and/or in the complicated context, is also a major concern. A potential approach to improving the stability is to coat the non-covalent assemblies with protective materials, such as silica [230] or polymer composition, and the self-assembled dye aggregate nanotubes composited by polymeric scaffolds have been reported to show resiliency even under extreme high-temperature conditions [41]. Finally, there is a great challenge to design stimuli-responsive dye aggregates as dynamic imaging or therapy agents that their optical features can be manipulated by environmental cues for precise and smart treatments. DNA nanotechnology can provide ‘smart’ templates which can respond to environmental signals and actuate their spectral properties. Additionally, DNA can also serve as an excellent targeting moiety, such as a host of aptamers with ability to specifically recognize biological markers [231], and the exceptional therapeutics agents and/or carriers for gene therapy [232]. These properties can improve the performance of these imaging/therapeutic agents that serve as precise medicines with enhanced specificity and synergistic effects. Leveraging the advantages of multichromophore photonic complexes and DNA nanotechnology, we envision a bright future for biomimetic photonic materials and their broad applications in the field of energy and biomedicine.

## Abbreviations

LH1: Light-harvesting complex 1
LH2: Light-harvesting complex 2
BChl: Bacteriochlorophyll
NIR: Near infrared
BODIPY: Boron dipyrromethene
G4: G-quadruplexes
EPO: Eosinophil peroxidase
MPO: Myeloperoxidase
PA: Photoacoustic
SBR: Signal-to-background ratio
PTT: Photothermal therapy
PDT: Photodynamic therapy
ROS: Reactive oxygen species
ssDNA: Single-stranded DNA
dsDNA: Double-stranded DNA
FRET: Förster Resonance Energy Transfer
QD: Quantum dot
MB: Molecular beacon
AuNR: Gold nanorod
